# Extractionof *Lepidium apetalum* Seed Oil Using Supercritical Carbon Dioxide and Anti-Oxidant Activity of the Extracted Oil

**DOI:** 10.3390/molecules161210029

**Published:** 2011-12-05

**Authors:** Wei Xu, Kedan Chu, Huang Li, Lidian Chen, Yuqin Zhang, Xuchong Tang

**Affiliations:** 1 Pharmacy College of Fujian University of Traditional Chinese Medicine, Fuzhou 350108, China; 2 Institute of Chemical Technology of Hua Chiao University, Xiamen 361021, China

**Keywords:** *Lepidium apetalum*, response surface methodology, supercritical fluid extraction, GC-MS, anti-oxidant activity

## Abstract

The supercritical fluid extraction (SFE) of *Lepidium apetalum* seed oil and its anti-oxidant activity were studied. The SFE process was optimized using response surface methodology (RSM) with a central composite design (CCD). Independent variables, namely operating pressure, temperature, time and flow rate were evaluated. The maximumextraction of *Lepidium apetalum* seed oil by SFE-CO_2_ (about 36.3%) was obtained when SFE-CO_2_ extraction was carried out under the optimal conditions of 30.0 MPa of pressure, 70 °C of temperature, 120 min of extraction time and 25.95 L/h of flow rate. GC-MS analysis showed the presence of four fatty acids in *Lepidium apetalum* seed oil, with a high content (91.0%) of unsaturated fatty acid. The anti-oxidant activity of the oil was assessed by the 2,2-diphenyl-1-picrylhydrazyl (DPPH) radical-scavenging assay and 2,2′-azino- bis(3-ethylbenzthiazoline-6-sulphonic acid) diammonium salt (ABTS) test. *Lepidium apetalum* seed oil possessed a notable concentration-dependent antioxidant activity, with IC_50_ values of 1.00 and 3.75 mg/mL, respectively.

## Abbreviations

SFE: supercritical fluid extraction
RSM: response surface methodology
CCD: central composite design
DPPH: 2,2-diphenyl-1-picrylhydrazyl
ABTS: 2,2′-azino-bis(3-ethylbenzthiazoline-6-sulphonic acid) diammonium salt

## 1. Introduction

*Lepidium apetalum* seed is the dry mature seed of *Lepidium apetalum* Willd, which is a plant of the Brassicaceae family. In China it is mainly produced in Hebei and Liaoning provinces, Nei Monggol, *etc.*, and it is characterized by its acrid and bitter in taste and cold in nature. It has the ability to clear away heat from the lung and relieve asthma, promoting diuresis and detumescence [1]. It is used in folk medicine for the treatment of sputum and phlegm turbidity in lungs, coughs and abundant sputum, inflated chest, not lying, abdominal edema and urinary obstruction. Modern pharmacological studies have shown that *Lepidium apetalum* seed has cardiotonic, cholesterol level regulating, anti-pigmentation and oral cancer antagonism effects.

*Lepidium apetalum* seed has abundant oil, making it an important oil plant in China. The seed fatty oil content is 40%, and it is noted for its richness in unsaturated fatty acids, which can be as high as 70%. In addition *Lepidium apetalum* seed is also a rich source of flavonoids, sterols and cardiac glycosides [2,3]. These components have numerous physiological and therapeutic effects, such as antibacterial [2], cardiac [4], cholesterol level regulating [5] and anticancer properties [6]. In recent years it has mainly been used in the clinic for diseases such as heart failure, *cor pulmonale*, chronic nephritis, pneumonia, pigmentation, oral cancer and so on [7,8,9,10,11,12]. Moreover, people are increasingly turning their attention to *Lepidium apetalum* seed because of its abundant unsaturated fatty acids. The identification of novel natural anti-oxidants is one of objectives of this investigation due to their safety, effectiveness and low cost [13]. *Lepidium apetalum* seed oil has notable anti-oxidant activity as a natural anti-oxidant, making it an important resource to develop and take advantage of. This study’s objectives were thus to investigate the process of oil extraction from *Lepidium apetalum* seed and to examine its anti-oxidant activity.

Generally, the traditional methods of extraction of *Lepidium apetalum* seed oil include expeller pressing and conventional organic solvent extraction methods, *etc.* The yield of the former method is lower. The latter method as the drawback that the oil must be heated to distill it and contains residual solvents, and at the same time the oil is oxidatively unstable, and is easily subject to rancidity during the separation process [14]. Supercritical fluid extraction (SFE) is a new extraction and separation technique in food industries which has been widely applied [15,16]. SFE overcomes the defects of the conventional organic solvent extraction and expeller pressing methods. It is an excellent extraction that has numerous advantages such as lower operating temperature, good selectivity, one step from the extraction to the separation and avoidance of residual solvents [14]. It had been applied to bio-medicines, foods and other fields [17,18], especially for extracting lipids [19]. Supercritical CO_2_ fluid extraction (SFE-CO_2_) is the most commonly used SFE variant. CO_2_ is an inert gas, which is stable, nonflammable, non-corrosiveness, non-toxic, cheap, and easy eliminated from the extracts. Furthermore, SFE-CO_2_ needs lower critical pressures and temperatures. In the supercritical state, CO_2_ is in an intermediate phase between gas and liquid, and has polarity similar to liquid pentane and has excellent dissolving power for lipophilic compounds. SFE-CO_2_ had been used to extract many plant components, but to the best of our knowledge no extraction of *Lepidium apetalum* by SFE-CO_2_ has been reported.

Response surface methodology (RSM) is a relatively new method of optimization of experimental conditions. It is suited for solving nonlinear data processing issues. RSM considers random error and fits once or twice with a simple polynomial model of the unknown complicated function. It has many advantages like relatively simple calculation, less number of experiments, short cycle and regression equations with high accuracy. It is a effective method to reduce development costs, optimize processing conditions, improve product quality, and solve practical problems in the production process by analyzing the resulting graphs. While the traditional methods of mathematical statistics often employ orthogonal or uniform designs that can simultaneously consider several factors to optimize factor levels, they cannot find the regression equation based on the given factors of the entire region between the values and the response function to choose the best combination. Nowadays RSM is increasingly used to optimize the conditions of experiment methods, such as ultrasonic extraction, SFE, microwave extraction, *etc.* [20,21,22].

This study used SFE to extract *Lepidium apetalum* seed, and optimized the main parameters (extraction pressure, extraction temperature, extraction time and CO_2_ flow rate) by a Central Composite Design RSM with the help of the experimental design software Design-Expert 7.1.3 Trial version, which was based on single factor experiments. GC-MS was used to analyse the chemical composition of *Lepidium apetalum* seed, and the study also determined the anti-oxidant activity of the extracted oil by the 2,2-diphenyl-1-picrylhydrazyl (DPPH) radical-scavenging assay and 2,2′-azino-bis(3-ethyl- benzthiazoline-6-sulphonic acid) diammonium salt (ABTS) test. The overall aim of the study was to provide a theoretical basis for the commercial exploitation of *Lepidium apetalum* seed.

## 2. Results and Discussion

### 2.1. Model Fitting and Significance Test

Four key parameters, namely extraction time, extraction pressure, extraction temperature and carbon dioxide flow rate were chosen to study the extraction process using the CCD design. The extraction yields of *Lepidium apetalum* seed oil obtained under the thirty different testing conditions are shown in Table 1.

**Table 1:** Experimental program and results for SFE-CO_2_ of *Lepidium apetalum* seed oil.

No.	A	B	C	D	Pressure	Temperature	Time	Flow	Extraction
	Pressure	Temperature	Time	Flow rate	MPa	°C	min	rate L/h	yield (%)
1	0	0	0	0	25	60	90	25	29.92
2	1	−1	1	1	30	50	120	30	32.58
3	1	−1	1	−1	30	50	120	20	31.17
4	1	−1	−1	1	30	50	60	30	29.47
5	0	0	0	−2	25	60	90	15	28.09
6	1	1	−1	1	30	70	60	30	30.53
7	1	1	1	−1	30	70	120	20	34.42
8	−1	−1	−1	−1	20	50	60	20	25.01
9	−1	1	−1	−1	20	50	60	20	15.21
10	0	−2	0	0	25	60	60	30	30.11
11	−1	−1	1	−1	20	50	90	20	28
12	0	0	−2	0	25	60	30	25	25.13
13	0	0	0	0	25	60	90	25	31.97
14	0	0	0	0	25	60	90	25	33.06
15	0	0	0	0	25	60	90	25	29.52
16	−1	1	1	−1	20	70	120	20	27.07
17	−1	1	1	1	20	70	120	30	27.54
18	−1	−1	−1	1	20	50	60	30	28.33
19	2	0	0	0	35	60	90	25	30.32
20	0	0	0	0	20	60	90	25	33.05
21	−2	0	0	0	15	60	90	25	11.79
22	1	1	1	1	30	70	120	30	35.56
23	−1	1	−1	1	20	70	60	30	17.44
24	0	0	0	2	25	60	90	35	30.66
25	0	2	0	0	25	80	90	25	31.82
26	0	0	2	0	25	60	150	25	30.18
27	1	−1	−1	−1	30	50	60	20	31.06
28	0	0	0	0	25	60	90	25	31.98
29	1	1	−1	−1	30	70	60	20	30.73
30	−1	−1	1	1	20	50	120	30	28.15

Multiple regression analysis was done for the data of the yields of *Lepidium apetalum* seed oil in Table 1 using the Design-Expert 7.1.3 Trial software. The result of the test statistics (F-test and probability) are shown in Table 2. The quadratic multiple regression equation of independent variables was:

Extraction yield = −9.80552 + 4.35796A − 1.45006B + 0.059618B + 1.20396D + 0.036487AB − 5.34583E − 003AC − 0.013525AD + 5.13542E − 003BC + 4.37500E − 004BD − 2.45833E − 004CD − 0.098587A^2^ + 1.28125E − 004B^2^ − 9.05208E − 004 C^2^ − 0.015388D^2^

where Extraction yield was the yield of *Lepidium apetalum* seed oil (%) and A, B, C and D were the actual values of the variables for extraction pressure (MPa), temperature (°C) ,time (min) and carbon dioxide flow rate (L/h) respectively. The Model F-value of 14.71 and the probability (P) value of 0.0001 (P < 0.01) imply that the model is significant. It demonstrated that there was good fit between the model and the actual experiment dataset and the linear relation between response value and variables was significant.

The "Lack of Fit F-value" of 1.90 and the probability (P) value of 0.2489 (P > 0.05) imply that the Lack of Fit is not significant relative to the pure error. R-Squared was 0.9321 and the "Pred R-Squared" of 0.6701 is in reasonable agreement with the "Adj R-Squared" of 0.8687, which measured the fitness of models, so the results indicatd that the model was accurate for predicting response variations. All in all the responses were explained well by the regression equation, and this allowed it to establish response surfaces and it was feasible to use the regression models to predict the the yields of *Lepidium apetalum* oil.

From Table 2, the investigated factors and their interactions significance were examined. First-order terms of various processing parameters (A,C) and the second-order terms of extraction pressure (A^2^) and time (C^2^) had high significance (P < 0.01), and this implied that pressure and time strongly impacted the yield of *Lepidium apetalum* seed oil. Pressure and temperature, temperature and time had high significance (P < 0.01), therefore the impact of the tested factors on the response value was not a simple linear relationship.

**Table 2:** *Lepidium apetalum* seed extraction yield the regression equation coefficient and significant testing.

Source	Sum of	df	Mean square	F-value	P-value	
	squares					
Model	766.95	14	54.78	14.716	<0.0001	significant
A-Pressure	382.64	1	382.64	102.71	<0.0001	
B-Temperature	5.85	1	5.85	1.57	0.2293	
C-Time	91.3	1	91.3	24.51	0.0002	
D-Flow	6.07	1	6.03	1.63	0.2212	
AB	53.25	1	53.25	14.29	0.0018	
AC	10.29	1	10.29	2.76	0.1173	
AD	1.83	1	1.83	0.49	0.4942	
BC	37.98	1	37.98	10.19	0.0061	
BD	7.656E−003	1	7.656E−003	2.055E−003	0.9644	
CD	0.022	1	0.022	5.840E−0.003	0.9401	
A2	166.62	1	166.62	44.73	<0.0001	
B2	4.503E−003	1	4.503E−003	1.209E−003	0.9727	
C2	18.2	1	18.2	4.89	0.043	
D2	4.06	1	4.06	1.09	0.3131	
Residual	55.88	15	3.73			
Lack of Fit	44.22	10	4.42	1.9	0.2489	not significant
Pure Error	11.66	5	2.33			
Cor Total	822.83	29				

Values of "Prob > F" less than 0.0500 indicate model terms are significant.

### 2.2. Optimization of the Lepidium apetalum Seed Oil Yield

Four key parameters, namely extraction time, extraction pressure, extraction temperature, and carbon dioxide flow rate were chosen to study the extraction process by CCD design based on the orthogonal test and preliminary experiment results.

**Figure 1:**
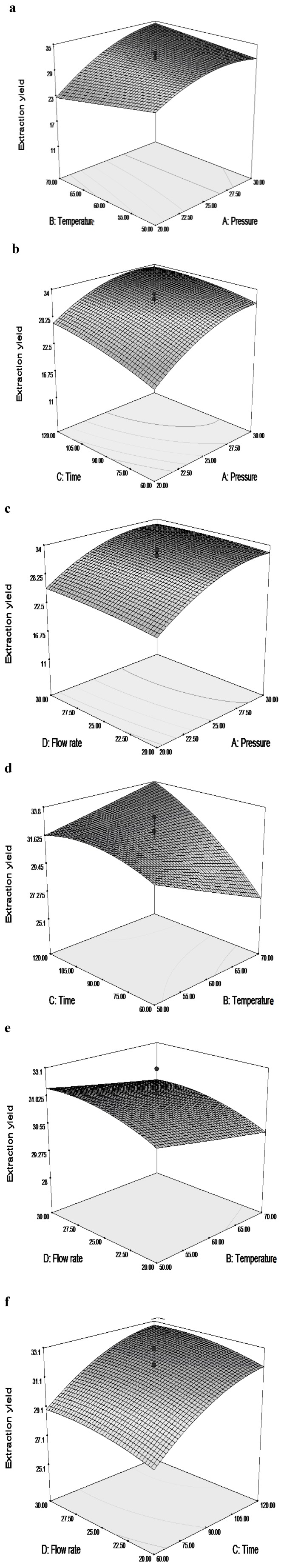
Response surfaces representations for the yield of oil (**a–f**) from *Lepidium apetalum* seed. (**a**) Effect of pressure (P) and temperature (T) under fixed flow rate (F) 25L/h and extraction time (t) 90 min; (**b**) Effect of P and t at F = 25 L/h and T = 60 °C; (**c**) Effect of F and P at T = 60 °C and t = 90 min; (**d**) Effect of T and t at P = 25 MPa and F = 25 L/h; (**e**) Effect of F and T at P = 25 MPa and t = 90 min; (**f**) Effect of F and t at P = 25 MPa and T = 60 °C.

RSM graphs can directly reflect the impact of factors on the response value, which were the extraction rate corresponding to factors A, B, C, D consisting of a three-dimensional response surface graph. Its interactions during the procedure could be found from the response surface graphs, shown in Figure 1. The graphs were plotted with two input variables kept constant at the zero-level value, and the other two varying over the experimental ranges. Figure 1 visualizes the response surface graph for the extraction yield of *Lepidium apetalum* seed corresponding to the factors extraction pressure, extraction temperature, extraction time and the flow rate of CO_2_. Figures **1****a–c** show the effects of pressure on the yield with each of the three other factors held constant. The yield increased to a maximum at a certain levels, then the yield changed little with continued increase of pressure. This phenomenon could be explained by the physical properties of solvent CO_2_, whose density increases with increasing pressure, which makes the ability to dissolve *Lepidium apetalum* seed oil increase. At the maximum of yield the pressure corresponds to the miscibility and solubility maximum pressures.

In Figures 1b,d,f, the graphs show the effects of time with each of the three other factors on the yield. The trend was similar to the pressure. It may be that interaction time of the seed and solvent CO_2_ increased and concentration of the oil increase. But when the time reached a certain level the solubility got saturation. The influence of the two other factors (temperature and flow rate) was not as significant as that of the pressure and time.

Optimization of extraction condition was achieved. It could be seen that the best extraction yield (36.3%) was reached at a pressure of 30 MPa, a temperature of 70 °C, a time of 120 min and flow rate of 25.95 L/h. A mean value of extraction yield 36.2% (n = 3) with a relative standard deviation (RSD) of 1.86% was obtained from actual experiments, which demonstrated the validation of the RSM model and good agreement with the predicted value, so we may conclude that the model is satisfactory and accurate.

### 2.3. Composition of Lepidium Apetalum Oil

**Figure 2:**
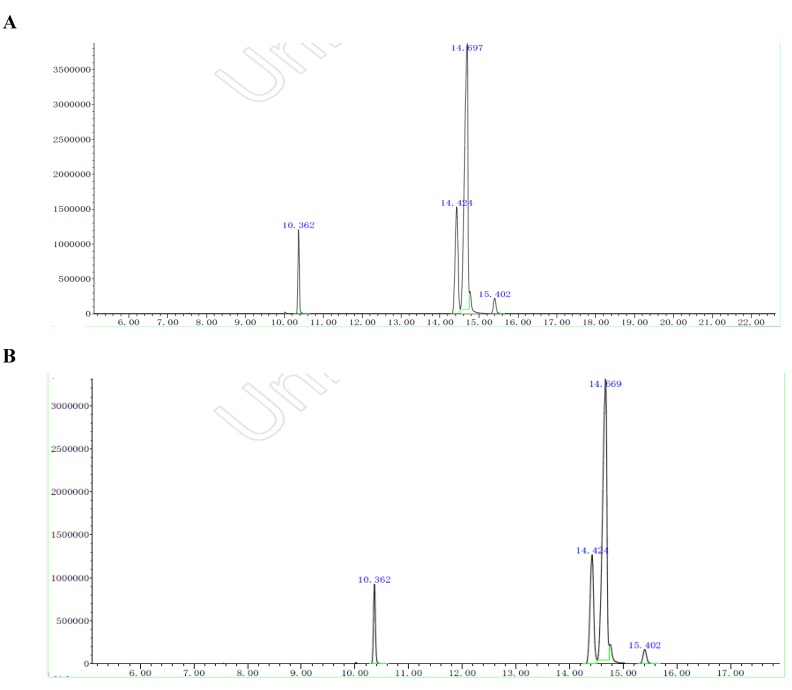
Total ion chromatogram (TIC) of the fatty acid methyl ester of *Lepidium apetalum* seed oil extracted by SFE-CO_2_ (**A**) and conventional organic solvent extraction (**B**).

Generally, the traditional methods of extraction of *Lepidium apetalum* seed oil include expeller pressing and conventional organic solvent extraction methods, but there have been no reports about extraction *Lepidium apetalum* seed by SFE-CO_2_. Now the two different extracting methods, conventional organic solvent extraction method and SFE-CO_2_ were employed to extract *Lepidium apetalum* seed oil, respectively. The chemical compounds of *Lepidium apetalum* oil were analyzed by GC-MS. The Total ion Chromatogram (TIC) of the fatty acid methyl ester of *Lepidium apetalum* seed oil extracted by SFE-CO_2_ is shown in Figure 2 and the contents of compounds were determined by using the normalization method. The four fatty acids were identified by computer searching the US States NIST27, NIST147 standard MS library, library matching and artificial analysis, and checking the relevant information according to the information obtained from GC-MS analysis. The fatty acid composition was: hexadecanoic acid (6.81% by SFE-CO_2_ extraction, 6.29% by conventional organic solvent extraction), 8,11-octadecadienoic acid (11.89% by SFE-CO_2_ extraction, 21.68% by conventional organic solvent extraction), 9,12,15-octadecatrienoic acid (79.09% by SFE-CO_2_ extraction, 70.25% by conventional organic solvent extraction), octadecanoic acid (2.29% by SFE-CO_2_ extraction, 2.20% by conventional organic solvent extraction). It could be seen that overall the contents of *Lepidium apetalum* seed oil compounds were similar by the two methods. The yield of *Lepidium apetalum* seed by SFE-CO_2_ was higher than by the conventional organic solvent extraction method except for 8,11-octadecadienoic acid, and unsaturated fatty acid content was enriched and very pure using SFE-CO_2_ compared with conventional organic solvent extraction. The reason for this may be that the oxygen content in the SFE-CO_2_ method was lower than that in conventional organic solvent extraction, as the SFE extraction is conducted almost without any oxygen. This condition protected the unsaturated fatty acids from oxidation. However, the raw material was exposed to much oxygen in the conventional organic solvent extraction, especially during the mixing process. So some unsaturated fatty acid was oxidation, which lead to the low content. The results showed that SFE-CO_2_ provides a safer, more efficient process than conventional organic solvent extraction, thus SFE-CO_2_ can be a better method to extract *Lepidium apetalum* oil.

*Lepidium apetalum* seed oil is rich in unsaturated fatty acid (91.0%). Unsaturated fatty acid is readily oxidized, therefore it must be prevented from contacting with air to avoid oxidation when it is stored. According to modern nutritional views, octadecenoic acid and octadecadienoic acid cannot be synthetized by the body itself, and must be absorbed from foods. They are essential fatty acids and are important to the body. They have effects on reducing blood cholesterol, prevention and treatment of cardiovascular disease, inhibition of tumor metastasis, reducing the incidence of coronary heart disease and improving immunity. Hence *Lepidium apetalum* seed oil has better nutritional and health effects, and has great research and development value.

### 2.4. Anti-Oxidant Activity of Lepidium apetalum Oil

DPPH and ABTS are employed widely to evaluate the antioxidant capacity and to screen for antioxidants in Traditional Chinese Medicine. They are quick, simple and sensitive methods to evaluate the antioxidant capacity of plants. In this article research the antioxidant capacity of *Lepidium apetalum* seed oil by determined by the DPPH and ABTS methods. Inhibition percentage was used as an index to compare the anti-oxidant activity.

**Figure 3:**
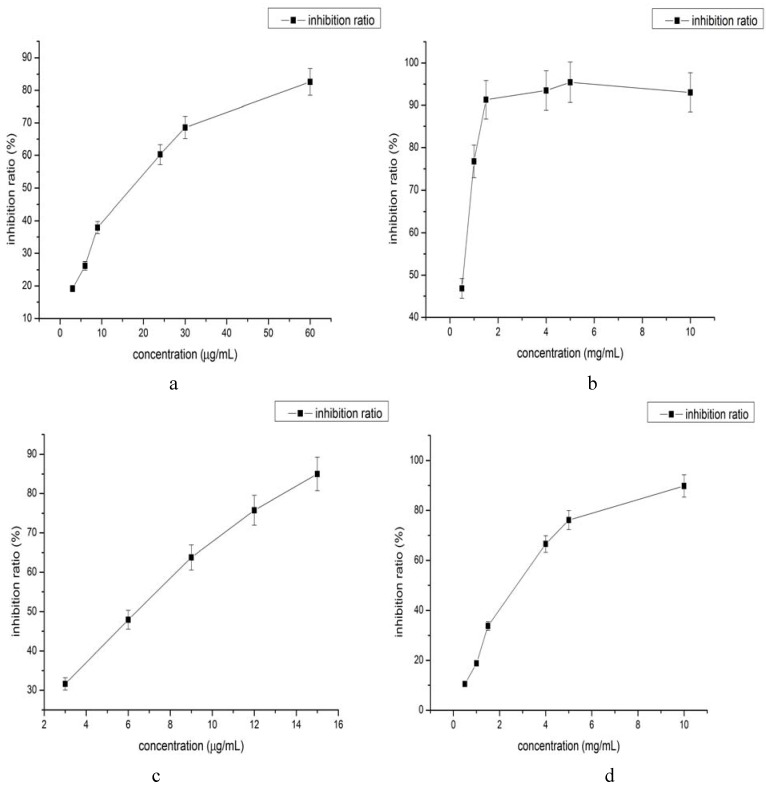
Antiradical activity determined using DPPH and ABTS. (**a**) The DPPH radical-scavenging of BHT. The concentration was from 3 µg/mL to 60 µg/mL; (**b**) The DPPH radical-scavenging of *Lepidium apetalum* seed oil. The concentration was from 0.5 mg/mL to 10 mg/mL. Data are averages with S.D. (error bars) from at least three independent experiments. * P < 0.05, *versus* control; (**c**) The ABTS radical-scavenging of BHT. The concentration was from 3 µg/mL to 60 µg/mL; (**d**) The ABTS radical-scavenging of *Lepidium apetalum* seed oil. The concentration was from 0.5 mg/mL to 10 mg/mL. Data are averages with S.D. (error bars) from at least three independent experiments. * P < 0.05, *versus* control.

DPPH is one of the most common methods to evaluate the free radical scavenging capacity. DPPH can absorb protonx and lose its chromophoric group to become yellow. Then it shows a maximum absorption at 515 nm for essential oil from *Lepidium apetalum*. The change of absorbance reflects the free radical scavenging capacity and allows us to evaluate the anti-oxidant activity. The different concentrated samples and BHT were assayed and the results of the DPPH radical-scavenging assay are shown in Figure 3a and Figure 3b. They show that *Lepidium apetalum* seed oil had a good anti-oxidant activity. The 50% reduction of the radical absorbance (IC_50_) was 1 mg/mL. The inhibition percentage for *Lepidium apetalum* seed oil ranged from 46.86% to 93.04% when the concentrations changed accordingly from 0.5 mg/mL to 10 mg/mL. It could be seen that *Lepidium apetalum* seed oil had the higher DPPH radical-scavenging ability. There was a good dose-response relationship between radical scavenging rate and the concentration of *Lepidium apetalum* seed oil. It also showed that when the concentration was 5 mg/mL the inhibition did not increase. This will help to choose the right concentration as antioxidants.

ABTS produces the blue-green cationic ion ABTS^+^ via activated oxygen oxidation. The reaction system fades when the sample that has anti-oxidant is added into ABTS, then the absorbance of free radical of ABTS is detected at the maximum absorption wavelength of 734 nm. The change of absorbance reflects the free radical scavenging capacity and allows evaluation of the anti-oxidant activity. The different concentrated samples and BHT were assayed and the results of the ABTS radical-scavenging assay are shown in Figure 3c and Figure 3d. They show that *Lepidium apetalum* seed oil had a good anti-oxidant activity. The 50% reduction of the radical absorbance (IC_50_) value was 3.75 mg/mL. The inhibition percentage for *Lepidium apetalum* seed oil ranged from 10.51% to 89.81% when the volumes changed from 0.5 mg/mL to 10 mg/mL. It could be seen that *Lepidium apetalum* seed oil had the higher ABTS radical-scavenging ability. There was a good dose-response relationship between radical scavenging rate and the concentration of *Lepidium apetalum* seed, namely the ABTS radical-scavenging rate increased with the increasing of the concentration of *Lepidium apetalum* seed.

Compared with BHT (IC_50_ by DPPH = 17.21 µg·mL^−1^, IC_50_ by ABTS = 6.25 µg·mL^−1^), the *Lepidium apetalum* seed oil revealed some lower activity, but *Lepidium apetalum* seed oil is only a crude extract, it still has important value for development as a component in food and medicine. Moreover, compare to the activities of other medicinal plants previously described in the literature such as *Ziziphora clinopodioides Lam.* and *Ziziphora pamiroalaica Juz* [23], *Elsholtzia rugulosa* [24], it has positive advantages.

Free radicals are molecules or ions with unpaired electrons. Excessive free radicals and their intermediate products injure biological membranes, enzymes, vitamins, proteins and the function of active cells seriously. It is important to find safe and high-efficiency anti-free radicals substances [25]. *Lepidium apetalum* seed oil is rich in unsaturated fatty acids (91.0%). It can defer senility and maintain health by lessening the toxic reactions of free radicals. So far the antioxidants most used in food are butylated hydroxylanisole (BHA) or dibutylated hydroxytoluene (BHT). Although they have good antioxidant properties, they have greater carcinogenic potential, so *Lepidium apetalum* seed oil could serve as a raw material to find ideal natural antioxidants.

## 3. Experimental

### 3.1. Materials and Reagents

High quality *Lepidium apetalum* seed produced in Heibei Province was procured from the Anhui Bozhou Chinese Medicine factory. Its identification was confirmed by Prof. Lu Wei, Fujian University of Traditional Chinese Medicine. Carbon dioxide (Purity > 99.5%) were obtained from Xiamen Rihong Gas Co., Ltd. Methanol, *n*-hexane, anhydrous sodium sulfate, KOH and DPPH, ABTS used in the assays were of analytical grade and were purchased from SCRC and Sigma.

### 3.3. Extraction of Lepidium Apetalum Oil

#### 3.3.1. Conventional Organic Solvent Extraction

A conventional hot reflux extraction method was performed to compare the extraction performance with SFE-CO_2_. *Lepidium apetalum* seeds were dried and sieved (40–60 mesh). Then approximately 100 g of sieved *Lepidium apetalum* seed and petroleum ether (60~90 °C) were added into a flask, which was connected to the extractor and condenser. After 4h some brown oil substances were extracted. Then 20% potassium hydroxide in ethanol (1:3, v/v) was added, heated and refluxed for 3 h. The extraction mixture was filtered, concentrated and cooled. Then it was extracted with ether (1:4.5, v/v) after adding a little water, the ether eluants were washed to neutrality and cleaned up with sodium carbonate solution. Finally after drying in 60 °C of an oven the oils obtained were weighed and the yields were calculated.

#### 3.3.2. *Lepidium Apetalum* Seed Oil Extraction Using SFE-CO_2_

An HA121-50-01 SFE device (Hua’an Supercritical Fluid Extraction Corp., Nan-tong, China) was used to extract *Lepidium apetalum* seed Oil. Operating steps were based on the method described by Zu *et al.* [26] with some modification. *Lepidium apetalum* seeds were dried for 10 h at 50 °C, and were ground in a rotary mill and then sieved (40–60 mesh). Then approximately 100 g of the the sieved *Lepidium apetalum* seed was loaded into a 1 L steel cylinder, then introduced into extractor II whilst pressure ring and gasket ring were installed, and the choke plug was tightened to process the extraction. When the scheduled temperature, pressure, time and the CO_2_ flow rate were achieved, the oil was obtained and collected in the separator. The amount of the extracted oil was accurately weighted and the extraction yield was determined.

#### 3.3.3. The Extraction Yield

The extraction yield was determined gravimetrically by the mass of extracted oil divided by the mass of *Lepidium apetalum* seed loaded in the extraction vessel, namely:

The extraction yield (%) = (mass of extracted oil / mass of dried material) × 100%

### 3.4. Experimental Design

CCD combined with RSM was employed to research and explore the key parameters in the extraction of *Lepidium apetalum* seed oil. The optimized parameters were determined. Four key parameters, namely extraction time, extraction pressure, extraction temperature and carbon dioxide flow rate at five different levels each, were employed based on the single factor experiments and pre-tests. The parameters chosen and their levels were based on preliminary experiments. According to the principles of experimental design four RSM parameters and five levels were employed. Every independent variable was projected on five levels. The levels and factors chosen for the trial are shown in Table 3.

**Table 3:** Level and factors chosen for the trial.

Variable	Code	Levels				
		−2	−1	0	1	2
Pressure (MPa) X_1_	A	15	20	25	30	35
Temperature (°C) X_2_	B	40	50	60	70	80
Time (min) X_3_	C	30	60	90	120	150
Flow (L/h) X_4_	D	15	20	25	30	35

The mathematical model was built on the basis of *Lepidium apetalum* seed oil extraction yield. A quadratic polynomial regression model assumed by the least squares method was:

Extraction yield = X_0_ + X_1_A + X_2_B + X_3_C + X_4_D + X_12_AB + X_13_AC + X_14_AD + X_23_BC + X_24_BD + X_34_CD + X_11_A^2^ + X_22_B^2^ + X_33_C^2^ + X_44_D^2^

where extraction yield is a response; X_0_ is a constant; X_1_,X_2_,X_3_ and X_4_ are linear coefficients; X_l2_, X_13_, X_14_, X_23_, X_24_ and X_34_ are cross-product coefficients; and X_11_, X_22_, X_33_ and X_44_ are quadratic coefficients. Experiments were conducted under the optimal extraction conditions to verify the validity of the experimental design. Three triplicate tests were performed.

### 3.5. GC-MS Analysis of Lepidium apetalum Fatty Oil

#### 3.5.1. Preparation of Fatty Acid Methyl Ester

The KOH-methanol methyl esterification method was employed directly. *Lepidium apetalum* seed oil (0.2 g) was added into a 10 mL test tube equipped with a stopper and mixed with 0.5 mol·mL^−1^ KOH-methanol solution (1 mL). Then, the test tube with the stopper was placed in a water bath at 40 °C, and shaken for 30 min. Then *n*-hexane (1 mL) was added and the mixture kept in a water bath at 20 °C and shaken for 10 min. After cooling water was added to 10 mL and the mixture was extracted for 1 min. A small portion of anhydrous sodium sulphate was added to the liquid supernatant that was obtained after centrifuging 10 min at 12,000 r·min^−1^ rotational speed. The filtered liquid was prepared for GC-MS sample by 100 times dilution.

#### 3.5.2. GC-MS Analysis Conditions

Gas chromatograph conditions: DB-5MS Fused-Silica Capillary Column (30 m × 0.25 mm × 0.25 um, Agilent, USA) was used. The carrier gas, nitrogen, flowed at the rate of 1.0 mL·min^−1^. The sample was injected with a sampling volume of 1.0 µL and a split ratio of 40:1. The inlet temperature was seat at 280 °C. The oven temperature was initially held at 150 °C for 2 min, and then from 150 to 200 °C at 12 °C·min^−1^, which was held for 3 min.

Mass spectrometry conditions: electron impact ionization (EI) mode was used as the ionization mode at 70 eV of ionization energy and the 230 °C of ion source temperature. Other chosen parameters were as follows: MS quadrupole temperature, 150 °C; the EI collector current, 34.6 µA; Interface temperature, 280 °C; resolution ratio, 0.1 Aum; solvent delay 5.0 min; mass scan range: 30–450 AMU.

### 3.6. Determination of Anti-Oxidant Activity

Anti-oxidant activity was determined using the DPPH and ABTS assays [27,28,29]. The anti-oxidant activity of the *Lepidium apetalum* seed oil extraction under the optimal conditions was determined. BHT was used as a standard antioxidant. All experiments were carried out in triplicate.

#### 3.6.1. DPPH Radical-Scavenging Assay

Radical scavenging activity was determined spectrophotometrically with the stable DPPH radical. DPPH solution (0.2 mmol·L^−1^, 2 mL) was mixed with the oils that were diluted in ethyl acetate ranging from 0.1 mL–2.0 mL. They were shaken sufficiently and kept in the dark for 30 min at 37 °C in a water bath, and then the decrease of absorbance was measured at 515 nm employing a UV-Vis spectrophotometer. Percentage inhibition of DPPH was used as the index of the radical-scavenging activities of samples; it was calculated according to the formula [30]:

Inhibition percentage = (A_0_ − A_s_) / A_0_ × 100%

where A_0_—Absorption of the blank sample, and As—Absorption of *Lepidium apetalum* seed oil.

#### 3.6.2. ABTS Radical-Scavenging Activity

ABTS radical-scavenging activity was also determined spectrophotometrically with ABTS^+^. ABTS^+^ was prepared by the reaction of 7 mM ABTS solution and 2.4 mM potassium persulphate solution in equal amounts that were allowed to react for 12–16 h at room temperature in the dark before use. The ABTS^+^ stock solution was diluted with absolute ethyl alcohol to the absorbance of 0.70 ± 0.005 at 734 nm before using. Then ABTS^+^ solution (0.1 mmol·L^−1^, 2 mL) was mixed with the oils that was diluted in ethyl acetate ranging from 0.1 mL–2.0 mL. The samples were adequately shaken and kept in the dark for 6 min in a 37 °C water bath, and then the decrease of absorbance was measured at 734 nm employing a UV-Vis spectrophotometer. The percentage of scavenging inhibition capacity of ABTS^+^ was calculated according to the same formula as described in the DPPH radical assay.

### 3.7. Statistical Analysis

The data statistical analysis was performed by regression models and analysis of variance (ANOVA) using Design-Expert 7.1.3 Trial software. Data are presented as mean values ± standard deviation. Origin 8.0 software was employed to analyse and plot the data.

## 4. Conclusion

RSM with CCD was found to be valuable and appropriate to optimize the SFE extraction conditions of *Lepidium apetalum* seeds. The optimal technological conditions of extraction were a pressure of 30 MPa, a temperature of 70 °C, a time of 120 min and a flow rate of 25.95 L/h. The anti-oxidant activity of the oil was assessed by the DPPH and ABTS assays. It possessed notable concentration-dependent antioxidant activity with IC_50_ values of 1.00 and 3.75 mg/mL, respectively. It provides references for the scale up, development and utilization of the nutritional properties of this oil in field of nutritive and health care and *Lepidium apetalum* seed can play an important role as a health-promoting anti-oxidant agent with economical potential for the pharmaceutical industry.
